# Factors associated with never treatment and acceptability of mass drug administration for the elimination of lymphatic filariasis in Guyana, 2021

**DOI:** 10.1371/journal.pgph.0001985

**Published:** 2024-04-25

**Authors:** Claudia Duguay, Reza A. Niles-Robin, Charles R. Thickstun, Horace Cox, Annastacia Sampson, Jean Seme-Fils Alexandre, Nathely Caleb-Mars, Charles W. Goss, Ana Morice, Ronaldo G. Carvalho Scholte, Alison Krentel

**Affiliations:** 1 School of Epidemiology and Public Health, University of Ottawa, Ottawa, Ontario, Canada; 2 Neglected Tropical Diseases Programme-Vector control Services, Ministry of Health, Georgetown, Guyana; 3 Bruyère Research Institute, Ottawa, Ontario, Canada; 4 Neglected, Tropical, and Vector Borne Diseases, Pan American Health Organization, Washington, DC, United States of America; 5 Division of Biostatistics, Washington University in St. Louis School of Medicine, St. Louis, Missouri, United States of America; CSIR-Indian Institute of Chemcial Technology, INDIA

## Abstract

Guyana remains one of four countries in the Americas endemic for lymphatic filariasis (LF). Elimination of LF requires repeated annual mass drug administration (MDA) with sufficient levels of coverage for success. This study assesses the acceptability and never treatment of LF MDA using data from a routine assessment survey in 2021. A subset of individuals, over 20 years of age (n = 2498), were selected to receive an expanded questionnaire to examine factors associated with acceptability and never treatment. Assessed factors include respondent demographics, knowledge, risk perceptions of LF, and opinions on the MDA programme. The majority (73%) of those with scores above the acceptability threshold (score ≥22.5) reported participating in MDA two or more times. Factors strongly and positively associated with scoring above the acceptability threshold include beliefs in importance of participation in MDA for their community (aOR = 2.8, 95%CI (1.1–7.2)), perception of importance of LF treatment (6.9 (3.2–14.7)), receiving treatment in 2021 (2.9 (1.5–5.4)), and the number of self-reported times taking treatment for LF (2.2 (1.1–4.4)). Ten percent of respondents participated in the MDA for the first time in 2021, while 15% reported never treatment during any round of LF MDA. Three factors were statistically associated with participation in MDA across the two levels of the models (level 1: took LF treatment once versus never, and level 2: took LF treatment twice versus never) included: 1) scoring above the acceptability threshold (aOR = 6.2, 95%CI(3.8–10.0)), 2) self-reported importance of participation in MDA for their community (7.1 (2.9–17.8)), and 3) personal beliefs that they should take LF treatment even if they are not sick (2.6 (1.7–3.9)). As Guyana moves closer to LF elimination, these results provide further insight and understanding into programmatic results and could inform further action following MDA activities—particularly if an approach is needed to address never treatment during MDA.

## Introduction

Lymphatic filariasis (LF) is a mosquito-borne disease characterized by a debilitating chronic buildup of interstitial fluid and swelling (lymphedema) caused through repeated infection of the lymphatic system by *Wuchereria bancrofti*, *Brugia malayi*, or *B*. *timori* parasites [1]. While the exact agent and vector are geographically diverse, the clinical presentation remains consistent: a dramatic engorgement and thickening of skin, generally in the legs or groin, that can cause impairment of mobility, increased infection risk, and psychosocial ostracization [2, 3].

Prior to the commencement of the Global Programme to Eliminate Lymphatic Filariasis (GPELF) in 2000, LF constituted a global burden of approximately 5 million disability-adjusted life years [4] and was considered by the World Health Organization (WHO) to be the second largest cause of disability in the world [5]. Seventeen countries have eliminated LF as a public health problem since the start of the GPELF, with seventy-two countries remaining endemic for LF in 2021, including four in the region of the Americas[6]. Current modelling suggests that LF infects an estimated fifty one million people globally as of 2018 [6]—roughly one quarter of the number infected just two decades earlier [7].

### Mass drug administration

WHO recommends the delivery of treatment through multiple rounds of mass drug administration (MDA) with effective coverage (≥65%) to at-risk and eligible populations, irrespective of infection status, to reduce transmission potential in endemic areas [8]. The current MDA drug regimens for areas where onchocerciasis is not co-endemic include diethylcarbamazine and albendazole (DA), used independently or in combination [9]. This regimen, however, has limited to no effect on adult filarial worms. Consequently, MDA efforts must be continued for at least 4–6 years to encompass the reproductive lifespan of the filarial worms [10]. The extended timeline of MDA may be challenged by shifting population dynamics, competing public health priorities, and the risk of decreasing interest and fatigue amongst endemic communities.

Recent studies suggest that annual MDA with a three-drug combination of ivermectin, diethylcarbamazine, and albendazole (IDA) is more effective than previous two-drug therapy (DA) in reducing microfilaria long term [11, 12] while maintaining low risk of adverse events in appropriate populations [13]. The magnitude of improvement IDA demonstrates in clearing microfilariae from those to whom it is administered, as well as a dramatic reduction in microfilariae resurgence, has led to the possibility of an accelerated timeline for LF elimination in some settings [8]. WHO accelerated the timeline for IDA approval at several stages [14], and recommended that IDA be used in settings where LF is not co-endemic with onchocerciasis or loiasis, where MDA has not yet begun, and where previous MDA efforts have not interrupted LF transmission [9].

Progress towards LF elimination is measured through a series of regular coverage evaluation surveys, surveys to measure antigenemia and microfilaria levels at sentinel and spot check sites, transmission assessment surveys, and surveillance. At each point along this pathway, there are opportunities to incorporate understanding about community acceptability of the MDA, including the measure of never treatment, to assess the effectiveness and equity of MDA.

### Acceptability

Acceptability is considered one of the four components of the highest attainable standard of health (availability, accessibility, acceptability, and quality) [15]. Acceptability can be defined as a “multi-faceted construct that reflects the extent to which people delivering or receiving a healthcare intervention consider it to be appropriate, based on anticipated or experienced cognitive and emotional responses to the intervention” [16]. Within the context of public health, increased use of an acceptability measure has been documented [17–23]. Within the context of MDA for LF elimination, an acceptability measure was developed and validated across multiple settings, including Guyana [17, 18]. These studies demonstrated LF MDA to be acceptable in the study populations across all settings, despite geographic area and likely exposure to the number of MDA rounds being the largest driver of variation in acceptability scores [17, 18]. The acceptability measure uses a 9-question scale to assess perceived relevance of the treatment, intention to take treatment, willingness to recommend others to take the treatment, beliefs on the effectiveness of the treatment and perceptions of importance to individual and community health [17]. Data from acceptability studies can help to understand low coverage and can be used to re-orient future MDA interventions to address gaps in knowledge and understanding about the LF treatment offered during MDA. This allows community perceptions to be considered and integrated into programme planning [24].

### Never treatment

Never Treatment is a term that refers to individuals who self-report to have never been treated for LF during any MDA round, representing a growing concern for elimination efforts [18, 25, 26]. Because the success of MDA relies on repeated rounds of preventive chemotherapy in an endemic population with effective coverage [27], individuals who are never treated risk becoming reservoirs for infection thereby stalling elimination efforts [26]. Understanding the characteristics of these individuals is essential for MDA programme development. Identifying those individuals and groups who may be systematically missed allows LF programmes to re-orient MDA activities to improve their reach as well as implement new social mobilization campaigns that adequately address concerns.

### Guyana

Guyana remains one of four countries in the Americas endemic for LF [6]. In October of 2019, Guyana joined the Pan American Health Organization Disease Elimination Initiative [28], to not only ensure the scale of non-communicable disease programmes, but to bolster an international effort to eliminate multiple communicable diseases, including LF. With an estimated 678,082 people requiring MDA for LF [6] and with preventive chemotherapy programmes since 2003 [29], new tools and approaches would be required to accelerate LF elimination in Guyana. Starting with a remapping survey in 2018–2019 to assess levels of LF endemicity, Guyana scaled their MDA programme to full geographic coverage of endemic areas in the six regions with highest levels of transmission (II, III, IV, V, VI, and X) and two focal areas within regions I and VII. In 2019, Guyana began administering IDA during MDA to accelerate elimination [30]. To reach the largest number of eligible participants, IDA MDA activities included a range of distribution approaches (door-to-door, distribution post, school, and work-based delivery) and tailored social mobilization campaigns to address regional differences identified in previous studies [18]. All treatments delivered during IDA MDA are taken under the direct observation of the distribution teams.

In this study, we aim to assess factors associated with never treatment during LF MDA and acceptability of LF treatment, following two rounds of IDA MDA in Guyana. Study results can potentially serve as a guiding framework to foster greater acceptance and participation in IDA MDA for LF elimination in Guyana.

## Methods

### Study design & population

This study was nested in an Epidemiological Monitoring Survey (EMS) following two rounds of IDA MDA in November 2021 in six of the eight endemic regions of Guyana. The primary EMS targeted 26 villages (or “hotspots”) with high levels of LF transmission and low IDA MDA coverage in both 2019 and 2021 and sampled 300 households in each hotspot following WHO guidelines for the monitoring and epidemiological assessment of MDA programmes [31]. An expanded Disease and Risk Perception (also called Acceptability) survey was administered to a subset of the respondents. One respondent per household in the targeted regions, above the age of 20, was randomly selected using Random UX [32] until 100 individuals had completed the survey in each hotspot (50 males and 50 females). When necessary to ensure parity of males and females in the acceptability sample, random samples within certain households were conducted preferentially from amongst only the underrepresented sex. Data was captured electronically using the ODK Collect Mobile Application and managed by Standard Data [33].

### Statistical analyses

The analysis focuses on two different and complimentary outcomes which are defined in the Supporting Information (S1 Table) and include: 1) acceptability of LF treatment and 2) never treatment (self-reported measure of never taking LF treatment over all MDA rounds). Acceptability of LF treatment is defined in Krentel *et al* [17]. Briefly, an acceptability score is derived using a series of nine questions with four possible responses each (disagree a lot, disagree, agree, and agree a lot). The total points awarded for each responded ranged from 9 to 36. In this analysis, acceptability of LF treatment is a dichotomous indicator, where those scoring ≥22.5 are above the acceptability threshold and those scoring <22.5 are below the acceptability threshold [17]. To derive levels of never treatment, a three-level metric was derived as the self-reported number of times a respondent has taken LF treatment with possible responses of never, one time, and two or more times.

Candidate explanatory variables hypothesized to be associated with acceptability and never treatment are defined in the Supporting Information (S1 Table) and include: 1) respondent demographics (sex, age, education, region), 2) knowledge about LF and risk perceptions (modes of transmission, concern about LF personally, perception of number of people in village with LF, belief that LF infection can be asymptomatic), 3) participation in the MDA of LF treatment (received and swallowed medication in 2021), 4) acceptability of the MDA of LF treatment (take treatment even if not sick, opinion about LF medicine), and 5) questions about MDA programme (importance of participation in MDA).

Frequency tables including chi-square tests were used to identify differences in the demographic profiles of the six regions of the study. Cells with fewer than 5 observations were collapsed—although “I don’t know” and “neutral” responses were always left as separate categories. The regions were also collapsed to encompass rural regions with a lower relative LF transmission (II, V, VI, X), a rural region with a higher relative LF transmission (III), and the capital region (IV). Participants with discrepant answers surrounding never treatment (i.e., stated that they swallowed treatment in 2021 and self-reported never taking LF treatment) were removed from the analysis. The analysis was conducted using SAS version 9.4 (SAS Institute, Cary, NC, USA) and *P-value*s less than 0.05 were considered statistically significant.

Univariable and multivariable mixed-effect logistic regression accounting for a clustered sampling design were conducted in RStudio version 4.1.3 (R Core Team, 2018) to explore factors associated with the acceptability of LF treatment. The “GLMER” function in the lme4 package in RStudio version 4.1.3 (R Core Team, 2018) was used to fit the generalized linear mixed model using the default Laplace approximation, a logit link, and random effects at the hotspot level (the smallest geographical clustering area) [34]. All candidate variables were entered in the multivariable model. All combinations of the model (2^n^ models, where n is the number of predictors in the full model) were fit using the Dredge function in the MuMIn package [35], and the model that minimized the Akaike information criterion (AIC) was selected. The final model based on minimizing the AIC, was fit and interpreted.

Univariable and multivariable multinomial mixed-effect logistic regression accounting for a clustered sampling design were conducted in RStudio version 4.1.3 (R Core Team, 2018) to explore factors associated with never treatment. The reference for this outcome was respondents that self-reported never taking LF treatment, and the two comparison groups included those that self-reported taking LF medicine once and twice (level 1: those self-reporting taking LF medicine once compared to those never treated, and level 2: those self-reporting taking LF medicine two or more times compared to those never treated). The “GLMER” function in the lme4 package in RStudio version 4.1.3 (R Core Team, 2018) was used to fit the generalized linear mixed model using the default Laplace approximation, a logit link, and random effects at the hotspot level [34]. All candidate variables were entered in the multivariable model to assess in what ways the hypothesized factors associated with never treatment converged and diverged at the different levels of participation in the LF MDA (never treated, taken once, or taken twice).

### Ethics statement

Research ethics was approved through the Ministry of Health, Guyana (IRB #82/2021) and reviewed with exemption by the Pan American Health Organization (PAHOERC.0432.01). All participants in this study were adults aged 20 years or older. Each participant was read an informed consent form by data collectors, which was read and signed by each participant prior to commencing data collection. Participants were provided with a copy of the informed consent form for their records and contact details for the study coordinator and the President of the Ethics in Research Committee of the Ministry of Health, Guyana if they wished to convey any questions or concerns with the conduct of the study. Additional information regarding the ethical, cultural, and scientific considerations specific to inclusivity in global research is included in the Supporting Information (S1 Text).

## Results

### Characteristics of the study population

A total of 2498 adults were included in this analysis. Overall, the sex of the study respondents was equally distributed: males (49%, 1215/2498) and females (51%, 1283/2498). There were statistically significant differences (*P-value*s <0.05) in the demographic profiles (except for sex) of all six regions. Most of the respondents completed primary top school (45%) with some regional differences—Region V completing the least education (none or primary school: 50%) and Region X completing the most education (tertiary or university: 22%). Acceptability also varied across the study regions with Region V having the greatest proportion of respondents above the acceptability threshold (97%) and Region II with the least (87%). Thirty-three (1.2%) respondents had discrepant answers for never treatment and were removed from the analysis (Table 1).

**Table 1:** Demographic profile of the study participants by region (n = 2498).

	Total	Region					
		ii	iii	iv	v	vi	x
	n = 2498	n = 397	n = 367	n = 556	n = 257	n = 553	n = 368
Sex							
Female	1283(51%)	201(51%)	191 (52%)	297(53%)	131(51%)	288(52%)	175(48%)
Male	1215(49%)	196(49%)	176 (48%)	259(47%)	126 (49%))	265(48%)	193(52%)
Age *							
20–29	502(20%)	57(14%)	65 (18%)	100(18%)	69(27%)	135(24%)	76(21%)
30–39	438(18%)	70(18%)	71 (19%)	97(17%)	40(16%)	92(17%)	68(19%)
40–49	515(21%)	89(22%)	79 (22%)	107(19%)	45(18%)	99(18%)	96(26%)
50–59	487(20%)	85(21%)	71 (19%)	112(20%)	58(23%)	103(19%)	58(16%)
60–69	371(15%)	59(15%)	60 (16%)	91(16%)	26(10%)	95(17%)	40(11%)
70+	185(7%)	37(9%)	21 (6%)	49(9%)	19(7%)	29(5%)	30(8%)
Education *							
No school	876(35%)	144(36%)	141 (38%)	183(33%)	128(50%)	238(43%)	42(11%)
Primary	1123(45%)	170(43%)	162 (44%)	254(46%)	84(33%)	251(45%)	202(55%)
Secondary	244(10%)	56(14%)	16 (4%)	58(10%)	24(9%)	45(8%)	45(12%)
University	255(10%)	27(7%)	48 (13%)	61(11%)	21(8%)	19(3%)	79(22%)
Acceptability Threshold *							
Above	2294(92%)	347(87%)	347 (95%)	528(95%)	248(97%)	490(89%)	334(91%)
Below	204(8%)	50(13%)	20 (5%)	28(5%)	9(3%)	63(11%)	34(9%)
Number of times taken medicine for LF *							
Never	379(15%)	93(23%)	46 (13%)	72(13%)	10(4%)	89(16%)	69(19%)
One time	373(15%)	61(15%)	67 (18%)	110(20%)	26(10%)	50(9%)	59(16%)
2 or more times	1746(70%)	243(61%)	254 (70%)	374(67%)	221(86%)	414(75%)	240(65%)

**Abbreviations:** LF: Lymphatic filariasis

Data are displayed as n (%)

* *P*<0.05 (significant differences between the six regions, by using the chi-squared test for comparison of proportions)

### Factors associated with acceptability

The distribution of acceptability scores in this study was non-normal and skewed to the left, with most of the respondents scoring above the acceptability threshold for LF treatment (92%, 2294/2498) (S2 Table, S1 Fig). Although the acceptability threshold is set at 22.5, the median acceptability score in this study was 27 (Inter-Quartile Range = 1) with nearly a fourth (39%) of the participants scoring a 3 (agree with the statement) on all nine acceptability questions. Ten of the eighteen candidate factors were included in the reduced multivariable model that assessed factors associated with acceptability of LF treatment. Strong and statistically significant indicators of acceptability of LF treatment were beliefs in importance of participation in the MDA programme for their community (important vs not important; Adjusted Odds Ratio (aOR) = 2.8; *P =* 0.001), perception of importance of LF treatment (important vs not important; aOR = 6.9; *P*<0.0001), receiving treatment in 2021 (aOR = 2.9; *P =* 0.001), and the number of self-reported time taking treatment for LF (twice vs never; aOR = 2.2; *P =* 0.014). Further emphasizing factors associated with acceptability of LF MDA, the majority (96%) of those scoring above the acceptability threshold believed that participation in MDA programme for their community was important and nearly 75% self-reported taking LF treatment more than two times. Other, weakly associated, factors included water as a mechanism of transmission (*P =* 0.007), taking LF treatment if not sick (*P =* <0.0001), and other members of their household taking LF treatment at the last distribution (*P =* 0.002). Although included in the final multivariable model, the following variables were not significantly associated with acceptability of LF MDA: sex, disease is asymptomatic, and personal concern about LF (Table 2).

**Table 2:** Results of mixed effect logistic regression analysis of factors associated with acceptability of LF MDA (n = 2498).

	Above Acceptability Threshold				
	%	OR (95% CI)	*P-value*	aOR (95% CI) *	*P-value*
Region					
iv	23	REF	-	-	-
iii	15	0.9 (0.3–2.6)	0.862	-	-
ii, v, vi, X	62	0.8 (0.5–1.2)	0.112	-	-
Sex					
Male	48	REF	-	REF	-
Female	52	1.5 (1.1–2.0)	0.008	1.4 (0.9–2.0)	0.174
Age					
20–29	20	REF	-	-	-
30–39	18	1.9 (1.1–3.1)	0.016	-	-
40–49	21	1.2 (0.8–1.8)	0.439	-	-
50–59	20	1.2 (0.8–1.9)	0.346	-	-
60–69	15	1.4 (0.8–2.2)	0.131	-	-
70+	7	0.9 (0.5–1.5)	0.941	-	-
Education					
No school	35	REF	-	-	-
Primary	46	1.0 (0.6–1.6)	0.869	-	-
Secondary	10	1.5 (1.1–2.1)	0.008	-	-
University	10	1.3 (0.8–2.2)	0.237	-	-
Number of times taken medicine for LF					
Never	12	REF	-	REF	-
One time	15	7.2 (4.4–11.8)	< .0001	1.8 (0.9–3.5)	0.075
2 or more times	73	16.3 (11.5–3.6)	< .0001	2.2 (1.1–4.4)	0.014
Importance of participation in MDA programme for community					
Not important	1	REF	-	REF	-
Neutral	20	2.5 (1.3–4.8)	0.002	1.6 (0.6–4.0)	0.171
Important	77	15.4 (8.0–29.7)	< .0001	2.8 (1.1–7.2)	0.001
Don’t know	2	0.5 (0.2–1.1)	0.145	0.6 (0.2–1.7)	0.373
Self-rated understanding of LF					
No knowledge	16	REF	-	-	-
A little	45	1.4 (0.9–2.0)	0.119	-	-
Average	20	1.5 (0.9–2.4)	0.118	-	-
Good	16	1.3 (0.8–2.2)	0.776	-	-
Very good	3	1.6 (0.7–3.9)	0.472	-	-
Mechanism of transmission: worms					
No	51	REF	-	-	-
Yes	49	1.8 (1.4–2.5)	0.003	-	-
Mechanism of transmission: mosquitoes					
No	11	REF	-	-	-
Yes	89	2.1 (1.4–3.0)	< .0001	-	-
Mechanism of transmission: water					
No	57	REF	-	REF	-
Yes	43	2.5 (1.8–3.4)	< .0001	1.7 (1.1–2.6)	0.007
Mechanism of transmission: don’t know					
No	86	REF	-	-	-
Yes	14	1.0 (0.7–1.6)	0.711	-	-
Believes LF to be asymptomatic					
Yes	48	REF	-	REF	-
Maybe	29	0.6 (0.4–0.9)	0.012	1.5 (0.9–2.4)	0.253
No	8	0.4 (0.2–0.6)	< .0001	0.6 (0.3–1.1)	0.069
Don’t know	16	0.3 (0.2–0.5)	< .0001	1.2 (0.7–2.1)	0.422
Perception of number of people in village with LF					
None	41	REF	-	-	-
Any	24	1.2 (0.8–1.8)	0.694	-	-
Don’t know	34	0.7 (0.5–1.0)	0.028	-	-
Personal concern about LF					
No, not at all	5	REF	-	REF	-
No, not really	13	0.8 (0.4–1.4)	0.271	0.4 (0.2–0.9)	0.018
Maybe	14	1.0 (0.6–2.0)	0.568	0.6 (0.3–1.5)	0.523
Yes, a bit	30	2.0 (1.1–3.7)	0.036	0.8 (0.3–1.7)	0.899
Yes, definitely	34	4.8 (2.4–9.8)	< .0001	1.1 (0.5–2.7)	0.314
Don’t know	4	0.4 (0.2–0.7)	0.010	0.4 (0.2–1.0)	0.063
Which is true about LF medicine					
Not important for health	2	REF	-	REF	-
Neutral	20	3.5 (2.0–5.9)	< .0001	2.8 (1.3–6.0)	0.007
Important for health	75	22.3 (12.8–8.9)	< .0001	6.9 (3.2–14.7)	< .0001
Don’t know	2	0.9 (0.5–1.7)	0.999	1.5 (0.6–3.8)	0.383
Take LF pills even if not sick					
No	17	REF	-	REF	-
Yes	62	8.7 (5.7–13.1)	< .0001	2.9 (1.8–4.7)	< .0001
Don’t know	22	1.5 (1.0–2.2)	0.134	1.8 (1.1–2.9)	0.038
Received medicine during 2021 distribution					
No	18	REF	-	REF	-
Yes	82	9.3 (6.8–12.8)	< .0001	2.9 (1.5–5.4)	0.001
Other members took LF medicine at same time					
No	17	REF	-	REF	-
Yes	70	5.9 (4.2–8.2)	< .0001	1.6 (1.0–2.5)	0.011
Don’t know	13	2.7 (1.7–4.3)	< .0001	2.4 (1.3–4.3)	0.002

**Abbreviations:** MDA: Mass Drug Administration, LF: Lymphatic filariasis, AOR: Adjusted Odds Ratio, OR: Odds Ratio

*Full models adjusted for hotspot number

### Factors associated with never treatment

More than three quarters (77%, 1909/2465) of the respondents received and swallowed the LF medicine in the last distribution. During any MDA round, most respondents (70%) self-reported taking LF treatment two or more times and 15% self-reported never taking LF treatment. Socioeconomic factors were not statistically associated with never treatment—however, the participants that self-reported never treatment are predominantly men (55%), those in the 20–29 age group (24%), and those who completed primary top school (45%). There are three factors that are statistically associated with never treatment across the two levels of the models (level 1: those self-reporting taking LF medicine once compared to those never treated, and level 2: those self-reporting taking LF medicine two or more times compared to those never treated) which include: 1) scoring above the acceptability threshold, 2) self-reported importance of participation in MDA programme for their community, and 3) personal beliefs that they should take the LF treatment even if they are not sick. A single factor is associated with level 1 and not level 2 of the model: personal concerns about LF medicine (level 1: *P =* 0.0165; and level 2: *P =* 0.0633). Two factors are associated with level 2 and not level 1 of the model:1) stating that LF was transmitted by worms (level 1: *P =* 0.5414; and level 2: *P =* 0.0268), and 2) self-reported importance of LF medicine (level 1: *P =* 0.4226; and level 2: *P =* 0.0225). The remaining factors are not statistically associated with either level of the multinomial never treatment model which include: 1) region, 2) sex, 3) age, 4) education, 5) self-rated understanding of LF, 6) stating that LF was transmitted by mosquitoes or water, 7) personal beliefs that LF is asymptomatic, and 8) perception of the number of people in their village with LF (Table 3).

**Table 3:** Results of mixed effect multinomial logistic regression analysis of factors associated with LF never treatment (n = 2465).

	Once vs never treatment			Two or more vs never treatment		
	Level 1			Level 2		
	%	aOR (95% CI)	*P-value*	%	aOR (95% CI)	*P-value*
Region						
Iv	25	REF	-	21	REF	-
Iii	15	0.6 (0.3–1.1)	0.078	14	0.8 (0.3–1.8)	0.535
ii, v, vi, X	60	0.9 (0.6–1.6)	0.802	65	1.4 (0.7–2.8)	0.294
Sex						
Male	53	REF		48	REF	
Female	47	0.9 (0.6–1.3)	0.542	52	1.2 (0.9–1.6)	0.279
Age						
20–29	23	REF	-	19	REF	-
30–39	20	1.4 (0.8–2.5)	0.233	17	1.3 (0.8–2.1)	0.368
40–49	19	1.4 (0.8–2.5)	0.229	20	1.6 (1.0–2.7)	0.055
50–59	17	0.7 (0.4–1.2)	0.153	20	1.2 (0.8–2.0)	0.413
60–69	11	1.0 (0.5–1.9)	0.955	156	1.9 (1.1–3.5)	0.024
70+	9	0.9 (0.4–1.8)	0.696	7	0.8 (0.4–1.5)	0.430
Education						
No	32	REF	-	36	REF	-
Primary school	8	1.3 (0.6–2.6)	0.527	10	1.6 (0.9–2.8)	0.144
Secondary school	46	1.1 (0.7–1.7)	0.816	44	0.9 (0.6–1.4)	0.716
University	14	1.6 (0.8–3.1)	0.161	9	0.5 (0.3–1.0)	0.052
Acceptability Threshold						
Below	18	REF	-	9	REF	-
Above	82	4.0 (2.2–7.4)	< .0001	91	6.2 (3.8–10.0)	< .0001
Importance of participation in MDA programme for community						
Not important	5	REF	-	3	REF	-
Neutral	28	3.8(1.2–12.3)	0.029	18	3.6 (1.4–9.1)	0.007
Important	61	4.9 (1.5–15.7)	0.008	78	7.1 (2.9–17.8)	< .0001
Don’t know	6	0.2 (0.02–1.5)	0.107	1	0.8 (0.3–2.6)	0.744
Self-rated understanding of LF						
No knowledge	22	REF	-	16	REF	-
A little	47	0.7 (0.4–1.1)	0.157	44	1.4 (0.9–2.2)	0.150
Average	17	0.5 (0.3–1.0)	0.040	20	1.6 (0.9–2.7)	0.103
Good	13	0.6 (0.3–1.1)	0.105	15	1.6 (0.9–3.0)	0.137
Very good	2	0.3 (0.1–1.4)	0.119	4	1.7 (0.5–5.4)	0.369
Mechanism of transmission: worms						
No	63	REF	-	51	REF	-
Yes	37	1.2 (0.7–1.8)	0.541	49	1.6 (1.1–2.3)	0.027
Mechanism of transmission: mosquitoes						
No	14	REF	-	11	REF	-
Yes	86	0.9 (0.5–1.7)	0.748	89	0.9 (0.6–1.6)	0.782
Mechanism of transmission: water						
No	67	REF	-	58	REF	-
Yes	33	1.6 (1.0–2.5)	0.063	42	1.4 (0.9–2.1)	0.130
Mechanism of transmission: don’t know						
No	88	REF	-	85	REF	-
Yes	12	0.8 (0.4–1.5)	0.520	15	0.9 (0.5–1.5)	0.585
Believes LF to be asymptomatic						
Yes	42	REF	-	45	REF	-
Maybe	29	0.6 (0.5–1.4)	0.510	29	0.9 (0.6–1.3)	0.454
No	9	1.1 (0.5–2.2)	0.831	8	1.0 (0.5–1.8)	0.919
Don’t know	20	0.8 (0.4–1.3)	0.332	17	1.0 (0.6–1.6)	0.988
Perception of number of people in village with LF						
None	40	REF	-	41	REF	-
Any	20	1.2 (0.7–2.0)	0.474	24	1.5 (1.0–2.4)	0.062
Don’t know	40	0.9 (0.6–1.4)	0.687	3	1.2 (0.8–1.7)	0.467
Personal concern about LF						
Not at all	5	REF	-	6	REF	-
No	19	2.1 (0.8–5.6)	0.124	14	1.0 (0.5–2.1)	0.992
Maybe	14	1.8 (0.7–4.9)	0.266	15	1.5 (0.7–3.1)	0.320
Yes	31	2.6 (1.0–6.6)	0.041	28	1.3 (0.7–2.7)	0.429
Definitely	25	3.3 (1.2–8.5)	0.017	32	2.0 (1.0–4.3)	0.063
Don’t know	6	2.3 (0.7–7.9)	0.187	5	2.0 (0.8–5.3)	0.127
Which is true about LF medicine						
Not important for health	5	REF	-	3	REF	-
Neutral	26	0.7 (0.3–1.9)	0.538	22	1.4 (0.6–3.2)	0.414
Important for health	58	1.5 (0.6–3.9)	0.423	71	2.6 (1.1–5.8)	0.023
Don’t know	10	0.8 (0.3–2.5)	0.731	3	0.3 (0.1–0.8)	0.022
Take LF pills even if not sick						
No	27	REF	-	18	REF	-
Yes	47	2.1 (1.3–3.3)	0.002	59	2.6 (1.7–3.9)	< .0001
Don’t know	25	1.2 (0.7–2.0)	0.589	23	1.3 (0.9–2.0)	0.216

**Abbreviations:** MDA: Mass Drug Administration, LF: Lymphatic filariasis

## Discussion

In this study, we identified factors associated with acceptability and never treatment using mixed-effect logistic and multinomial regression models. Scoring above the acceptability threshold was strongly associated with the number of times respondents reported taking LF treatment, although nearly ten percent of those that self-reported never treatment scored above the acceptability threshold. This study highlights the importance of emphasizing the message of participation in MDA for the benefit of community health, as this was highly associated with MDA participation and acceptability in our models. We also found that knowledge of the disease (self-reported understanding of LF) is not strongly associated with acceptability which is important to note when planning approaches to address never treatment, non-participation in the most recent distribution, or scores below the acceptability threshold [18, 36].

Many of the factors associated with scoring above the acceptability threshold in this study suggest a link between acceptability and social norms. Accepting individuals were more likely to believe they should take the pills even if they were not sick, if they felt participation was important for community health and if they took the medicine at the same time as other family members. While they were more likely to feel the medicine delivered during MDA was important for their personal health than those who scored below the threshold, these individuals were not any more likely to have higher levels of personal concern about LF. Our earlier study in Guyana [18] showed a similar association with social norms and acceptability, this time to a greater extent.

In these analyses, identifying water as a significant risk factor for LF was a predictor of scoring above the acceptability threshold, as well as initial and repeated participation in MDA. While not directly a mechanism for transmission for LF, standing water remains a key component in the life cycle of *Culex*. *quinquefasciatus* [2], and an important risk factor for numerous other diseases affecting people living in the area [37]. Whether this association with increased participation in the MDA indicates a misattribution of disease risks communicated in other public health campaigns—or the association is simply a spurious significance—future studies should consider the influence of competing messaging campaigns or, if possible, include a more substantive qualitative component to investigate these associations.

Within the global LF programme, measures of treatment coverage to eliminate LF have focused on coverage reported by the programme or measured during single cross-sectional coverage evaluation surveys conducted during routine monitoring and evaluation [6]. While data on coverage is imperative to evaluate MDA programme performance, it may not include an understanding of individual behavior and perspectives as they relate to coverage. Within programmatic monitoring and evaluation, the inclusion of a metric on never treatment (whether due to missed MDA rounds, refusals, or unintentional reasons) in some routine survey tools allows programmes to identify if this is a potential barrier to LF elimination. To this end, recent research on never treatment has used the dichotomy of “Never/Ever” participant status in order to evaluate the characteristics of never treated individuals during MDA efforts [18].

This analysis takes an additional step in evaluating participants by the number of MDA rounds in which they have participated. Here we considered that there are three distinct groups of analysis: 1) those who have never taken MDA; 2) those who have taken MDA one time; and 3) those who participated in the MDA two or more times. While this distinction may not always be important—individuals may certainly miss rounds of treatment for benign reasons—understanding the differences between categories can inform programme managers on the success of new MDA measures and tailoring of social mobilization campaigns to encourage first time participation and emphasize the importance of continuing to take treatment throughout all rounds of MDA.

In our analysis, we found that respondents who reported taking MDA two or more times shared a similar pattern of knowledge and beliefs as those who have taken MDA once, when compared to those who had never taken. Our model identified similar significant relationships between these two groups and never treated individuals across our variables of interest, and the resulting levels of the model are quite uniform. The major difference between the two levels is the strength of association, with most predictors indicating higher odds of participation in the twice vs never level, compared to the same factors in the once vs never.

These similarities suggest a uniform rationale for participation in Guyana. Those who have participated in the MDA once have done so for the same reasons as those who have participated more than once. Because many factors in this model may change with exposure to social mobilization campaigns and interaction with MDA rounds, it is possible that a large component of first time MDA participants may have done so due to interaction with these efforts for the first time. Guyana has dramatically increased all efforts surrounding LF MDA in the past few years with elimination targets drawing closer [30, 38], increasing the likelihood that these campaigns have provided new perspectives on the MDA in between MDA rounds.

Where differences in significance do occur, they relate to the individual. Personal concern for LF and importance of participation in the MDA for personal health both have single categories with differences in significance between the two levels of the model. Those who identified participation as important or very important for personal health were significantly more likely to have participated two or more times, which is not mirrored in those who participated only once. Conversely, those who identified that they were somewhat personally concerned about LF were significantly more likely to have participated once—an association not carried through to those who participated two or more times.

### Limitations

This study has some limitations that should be noted. We found that nearly a fourth of the participants agreed with all nine acceptability questions which brought their total acceptability score to 27. These respondents did not significantly differ by gender, region, nor hotspot level, but the frequency of their similar responses caused a skew in the distribution of the outcome. Acceptability has historically been used as a continuous metric to assess factors associated with acceptability of LF MDA, however this study dichotomized the metric to identify individuals that were above and below the acceptability threshold to ensure the validity of the statistical methods of analysis, given the skewed distribution in this particular sample [18]. The cut point used for the outcome was the mean acceptability score (22.5) which is consistent with previous studies and indicates a mean score of 2.5 for each question where 1 = disagree a lot, 2 = disagree, 3 = agree, and 4 = agree a lot [17]. By dichotomizing the outcome, we effectively assessed factors associated with those that felt negatively about LF MDA, rather than the detailed differences with every increase in score. Dichotomizing acceptability has also been utilized in prior related research measuring the acceptability of indoor-residual spraying for the control of malaria which points to the usability and feasibility of dichotomizing acceptability in certain settings [22].

Another limitation is that cross-sectional surveys are generally administered during the day which has historically resulted in a sample heavily skewed towards women since they are more likely to be home at the time of the survey [18]. To mitigate a skewed distribution and to ensure gender parity, we evenly sampled men and women in this study. This study had an even distribution of females (52%) and males (48%), however, there were significant differences in the age distribution among the two genders, with significantly older men in the sample (≥50 years old). This could be attributed to younger men (<50 years old) being away from their house during the day to work which may result in an underrepresentation of this age group in the sample.

Finally, while this data is captured from a valuable sample of at-risk individuals in Guyana it should not be considered representative of the characteristics or opinions of the population of Guyana as a whole as the EMS, in which this study was nested, specifically targets hotspots in endemic areas.

## Conclusion

This study assessed factors associated with never treatment and acceptability of MDA for the elimination of LF in Guyana in 2021. We found that factors strongly associated with scoring above the acceptability threshold include beliefs in importance of participation in the MDA for their community, perception of importance of LF treatment, receiving treatment in 2021, and the number of self-reported time taking treatment for LF. This study also found that factors statistically associated with never treatment across the two levels of the models include scoring above the acceptability threshold, self-reported importance of participation in MDA for their community, and personal beliefs that they should take the LF treatment even if they are not sick. These results can inform subsequent MDAs in Guyana, or elsewhere. These results and metrics (acceptability and never treatment) can be used to provide insight and tailor IDA MDA activities in Guyana, or other countries that are endemic for LF, particularly if an approach is needed to address never treatment.

Following the culmination of the 2021 EMS, Guyana engaged with stakeholders and partners in multiple discussions regarding the next steps towards LF elimination. Given the success of the two rounds of IDA MDA, it was determined that further rounds were not immediately necessary, and the country should move into a post-MDA surveillance phase. As of 2023, Guyana implemented its first IDA Impact Survey (IIS) in all endemic EUs. If subsequent MDA rounds are required, we suggest that the Guyana MoH develop social mobilization campaigns informed by this research to increase acceptability and participation in MDA. Specifically, this study provides specific evidence related to never treatment, and what factors are associated with participation once and participation two or more times. In particular, core themes for tailored social mobilization can highlight the importance of participation for community health and the importance of participating even though you are not sick are likely to encourage new and continued participation in MDA.

## Supporting information

S1 Fig Distribution of acceptability score. (DOCX)

S1 Table Variable definitions. (DOCX)

S2 Table Breakdown of similar answers for each acceptability question. (DOCX)

S1 Text Inclusivity in global research. (DOCX)
