# Supplementary figures and images for: Factors associated with never treatment and acceptability of mass drug administration for the elimination of lymphatic filariasis in Guyana, 2021
Source: PLOS Glob Public Health. 2024 Apr 25;4(4):e0001985. doi: 10.1371/journal.pgph.0001985 (PMC11045083; doi:10.1371/journal.pgph.0001985)

**S1 Fig**  Distribution of Acceptability Score


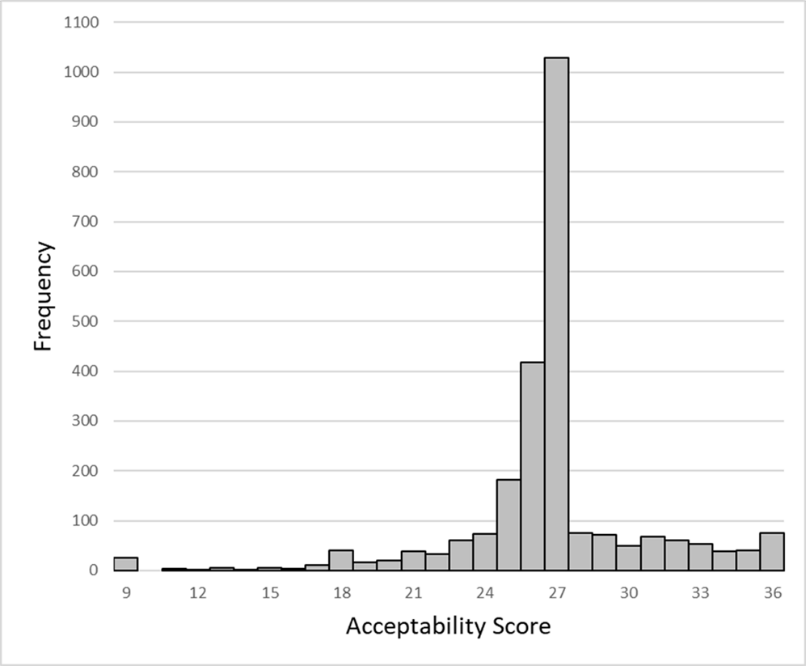

Supplement: S1 Fig — (DOCX) [file pgph.0001985.s001.docx]
