# Supplementary material for: Factors associated with never treatment and acceptability of mass drug administration for the elimination of lymphatic filariasis in Guyana, 2021
Source: PLOS Glob Public Health. 2024 Apr 25;4(4):e0001985. doi: 10.1371/journal.pgph.0001985 (PMC11045083; doi:10.1371/journal.pgph.0001985)
Supplement: S1 Table — (DOCX) [file pgph.0001985.s002.docx]

**S1 Table. Variable Definition**

| **Variable** | **Variable Definition** |
| --- | --- |
| Acceptability | **Levels:**   - Above threshold - Below threshold   **Definition:**  An acceptability score is derived using a series of nine questions with four possible responses each (disagree a lot, disagree, agree, and agree a lot). The total points awarded for each responded ranged from 9 to 36. Those scoring between ≥22.5 are above the acceptability threshold and those scoring <22.5 are below the acceptability threshold |
| Never Treatment | **Levels:**   - Never - Once - Two or more   **Definition**:  Self-reported number of times a respondent has taken LF treatment |
| Region | **Levels:**   - IV - III - II, V, VI, X   **Definition**:  These are pre-determined region names in Guyana. Regions were collapsed to encompass rural regions with a lower relative LF transmission (II, V, VI, X), a rural region with a higher relative LF transmission (III), and the capital region (IV). |
| Sex | **Levels:**   - Male - Female |
| Age | **Levels:**   - 20-30 - 30-40 - 40-50 - 50-60 - 60-70 - 70+ |
| Education | **Levels:**   - No education - Primary - Secondary - University   **Definition**:  The highest level of education completed |
| Importance of participation in MDA programme for community | **Levels:**   - Not important (not important at all or not very important) - Neutral - Important (important or very important) - Don’t know   **Definition**:  How important is your participation in the MDA programme for your community. |
| Self-rated understanding of LF | **Levels:**   - No knowledge - A little - Average - Good - Very good   **Definition**:  How would you describe your understanding of LF? |
| Mechanism of transmission | **Levels:**   - Yes - No   **Definition:**  How do you think LF is transmitted from person to person? Options: mosquitoes, worms, water, curse, hereditary, other. |
| Believes LF to be asymptomatic | **Levels:**   - Yes - Maybe - No - Don’t know   **Definition**:  Can a person have LF in their body and not have any symptoms. |
| Perception of number of people in village with LF | **Levels:**   - None - Any (few, some, quite a lot, many) - Don’t know   **Definition**:  How many people in your village do you think have LF |
| Personal concern about LF | **Levels:**   - Not at all - Not really - Maybe - Yes, a bit - Yes, definitely - Don’t know   **Definition**:  Are you concerned about LF personally? |
| Which is true about LF medicine | **Levels:**   - Not important for health (not important for my health at all or not very important) - Neutral - Important for health (important or very important for my health) - Don’t know   **Definition**:  In your opinion, which is true about LF medicine? |
| Take LF pills even if not sick | **Levels:**   - Yes - No - Don’t know   **Definition**:  Do you think you should take this medicine even if you don’t feel sick? |
