# Supplementary material for: Factors associated with never treatment and acceptability of mass drug administration for the elimination of lymphatic filariasis in Guyana, 2021
Source: PLOS Glob Public Health. 2024 Apr 25;4(4):e0001985. doi: 10.1371/journal.pgph.0001985 (PMC11045083; doi:10.1371/journal.pgph.0001985)
Supplement: S2 Table — (DOCX) [file pgph.0001985.s003.docx]

**S2 Table**  Breakdown of respondents with the same answer for each acceptability question

|  | **n** | **%** |
| --- | --- | --- |
| All 1s (Disagree a lot) | 25 | 1.00% |
| All 2s (Disagree) | 37 | 1.48% |
| **All 3s (Agree)** | **981** | **39.27%** |
| All 4s (Agree a lot) | 75 | 3.00% |
| Mix of answers | 1380 | 55.24% |
